# Retraction Note: Long non-coding RNA MLLT4 antisense RNA 1 induces autophagy to inhibit tumorigenesis of cervical cancer through modulating the myosin-9/ATG14 axis

**DOI:** 10.1038/s41598-025-17248-y

**Published:** 2025-09-01

**Authors:** Tingting Zhang, Tiantian Ji, Zhao Duan, Yuanyuan Xue

**Affiliations:** Department of Gynecology, The Second Affiliated Hospital of Medical College of Xi’an Jiaotong University, Xi’an, China

Retraction of: *Scientific Reports* 10.1038/s41598-024-55644-y, published online 16 March 2024

The Editors have retracted this Article. After publication, concerns were raised regarding the data presented in Figures 1,2 and 4. Specifically,

• the *SQSTM1/Siha* gel slice in Figure 1A appears to overlap with the *SQSTM1/MLLT4-AS1* gel slice in Figure 4H;

• the *ACTB* gel slices in Figure 1A appear to overlap with the *ACTB* gel slice in Figure 5D;

• the *ACTB/Hela* gel slice in Figure 2 appears to overlap with the ACTB gel slice in Figure 4H.

• a portion of the *Control* panel in Figure 4A appears to overlap with a portion of the *Vector* panel in the same figure;

• portions of the *MLLT4-AS1* panel in Figure 4B appear to overlap with other portions of the same panel;

• a portion of the *Vector* panel in Figure 4G appears to overlap with another portion of the same panel;

• a portion of the *MLLT4-AS1* panel in Figure 4G appears to overlap with another portion of the same panel.

The Authors provided some of the original data, but this was insufficient to address the concerns raised. The Editors therefore no longer have confidence in the results and conclusions reported.

The Authors did not reply to correspondence from the Editors about this retraction.

